# A. V. M. A.

**Published:** 1902-07

**Authors:** 


### A. V. M. A.

We have been notified by Secretary Stewart that the programme for the Minneapolis meeting is nearing completion, and the following papers have been promised, with several more under consideration:

"The Veterinary Profession, Past, Present and Future," by Professor D. McEachran, of Montreal, Quebec. "External Ulcerative Ano-vulvitis of Cattle," by Dr. J. J. Repp, of Ames, Iowa. "So-called Contagious Ophthalmia of Cattle," by Dr. T. D. Hinebauch, of Fargo, North Dakota. "Hospital Management of Dogs," by Dr. Chas. E. Ellis, of St. Louis, Mo. "Serums and Serum Therapy," by Dr. E. A.A.Grange, of New York City. "The Relationship of Veterinary Science to the Medical Profession," by Dr. D. King Smith, of Toronto, Ontario. "Barrenness in Bovines," by Dr. Chas. Schmitt, of Dodgeville, Wis. "Poisonous Stock Foods," by Dr. N. S. Mayo, of Manhattan,

Kansas. "Malarial Fever in the Horse," by Dr. F. Torrance, of Winnipeg, Man. "Some Features of the Texas Fever Problem," by Dr. W. C. Rayen, of Nashville, Tennessee.

Titles of papers by the following members have not been ascertained: Dr. W. Horace Hoskins, of Philadelphia, Pa.; Dr. Leonard Pearson, of Philadelphia, Pa.; Dr. S. D. Brimhall, of St. Paul, Minn.; Dr. J. S. Anderson, of Seward, Neb.; Dr. W. L. Williams, of Ithaca, N. Y.; Dr. M. E. Knowles, of Helena, Mont.; Dr. C. A. Carey, of Auburn, Ala.; Dr. M. Jacob, of Knoxville, Tenn.; Dr. C. H. Howard, of Calumet, Mich.; Dr. R. P. Lyman, of Hartford, Conn.

The West Hotel has been selected as headquarters and the meetings of the association will be held in the assembly hall of the hotel. The local committee have secured rates from a number of hotels and private boarding houses near to the headquarters, and a list of these with address will be sent to all the members that they may arrange for quarters prior to the meeting. The fact that the Minnesota State Fair will be in progress at that time will make it necessary to engage apartments before arrival in order to make sure of them.

The railroads have granted a one and one-third fare transportation, on the certificate plan. Those living within the concessions of the State Fair may secure a better rate.

The local committee of arrangements are planning many special features for the entertainment of all who come and more especially the ladies. The numerous beautiful lakes and pleasure resorts in close proximity to the twin cities of the North offer many attractions, and particularly among these is Lake Minnetonka, upon the shores of which it is proposed to hold the banquet in which it is hoped that the ladies will have a part.

The new building for the Veterinary Department of the State University has been completed, and in this building there is a clinic room with an amphitheatre, and arrangements are about completed for a series of demonstrations of surgical procedures at the clinic to be held at the close of the meeting.

---

MR. W. W. DIXON, the Chicago representative of the well-known firm of E. R. Squibb & Sons, will accompany the members of the "Journal Special" from Chicago to Minneapolis. Mr.

Dixon is located at the Plaza, Chicago, where he purposes entertaining all those who are going by the "Journal Special" with a luncheon at the Hotel Plaza, and afford them a magnificent view of Lake Michigan and Lincoln Park.

Those going by the "Journal Special" will be privileged in going or coming to stop off at Niagara Falls for a stay of three or four days. This feature of the trip should attract all those who have never seen this wonderful feature of our country.

Drs. A. H. Baker and Joseph Hughes, of the Chicago Veterinary College, extend a very cordial invitation to those who anticipate attending the Minneapolis meeting, and who may remain in Chicago for a few hours, to visit the Chicago Veterinary College. Those going by the route selected by them are invited to dine with them en route to Minneapolis.

Dr. D. E. Salmon, Chief of the Bureau of Animal Industry, will be among those who will accompany the "Journal Special" to Minneapolis, Minnesota.

Dr. W. C. Holden, of Delphos, Ohio, will be among those on the "Journal Special."

One of the ways that some of the Eastern veterinarians are planning to reach Minneapolis is as follows : From Philadelphia to Buffalo and return ; Buffalo to Chicago by lake, returning by rail ; Chicago to Minneapolis, at an estimated cost of $130. Where two occupy the same berth the expense will be reduced ten dollars. The lake route affords 1000 miles on the water ; the time consumed thereby is three days and two nights. Those who go by this route will have to leave Buffalo on Tuesday, August 26th, in order to arrive in time for the opening of the convention. The one and one-third rate over the "Burlington Route" from Chicago will be afforded to all who use this line, up to and including the last day of the convention.

